# CT characteristics in pulmonary adenocarcinoma with epidermal growth factor receptor mutation

**DOI:** 10.1371/journal.pone.0182741

**Published:** 2017-09-26

**Authors:** Jing Zhao, Julien Dinkel, Arne Warth, Roland Penzel, Niels Reinmuth, Philipp Schnabel, Thomas Muley, Michael Meister, Heike Zabeck, Martin Steins, Jian-yong Yang, Qian Zhou, Heinz-Peter Schlemmer, Felix J. F. Herth, Hans-Ulrich Kauczor, Claus Peter Heussel

**Affiliations:** 1 Department of Diagnostic and Interventional Radiology, University Hospital of Heidelberg, Heidelberg, Germany; 2 Translational Lung Research Center Heidelberg (TLRC), Heidelberg, Germany; 3 Department of Diagnostic and Interventional Radiology, The First Affiliated Hospital of Sun Yat-Sen University, Guangzhou, Guangdong, China; 4 Institute of Pathology, University Hospital Heidelberg, Heidelberg, Germany; 5 Airway Center North (ARCN), LungenClinic Grosshansdorf GmbH, Großhansdorf, Germany; 6 Translational Research Unit, Thoraxklinik at University of Heidelberg, Heidelberg, Germany; 7 Department of Thoracic Surgery, Thoraxklinik at University of Heidelberg, Heidelberg, Germany; 8 Department of Thoracic Oncology, Thoraxklinik at University of Heidelberg, Heidelberg, Germany; 9 Clinical Trials Unit, The First Affiliated Hospital of Sun Yat-Sen University, Guangzhou, Guangdong, China; 10 Department of Radiology, German Cancer Research Center (dkfz), Heidelberg, Germany; 11 Department of Pneumology and Respiratory Critical Care Medicine, Thoraxklinik at University of Heidelberg, Heidelberg, Germany; Peking University People's Hospital, CHINA

## Abstract

Comprehensively investigate the association of CT morphology and clinical findings of adenocarcinoma with *EGFR* mutation status. Retrospectively included 282 patients who was pathologically proved as lung adenocarcinoma with known *EGFR* mutation status (mutations: 138 patients, female: 86, median age: 66 years; wildtype: 144 patients, female: 67, median age: 62 years) and their pre-treatment CT scans were analyzed. CT findings and clinical information were collected. Univariate and multivariable logistic regression analysis were performed. Adjusted for age, gender and smoking history of two groups, significantly more patients with pleural tags, pleural and liver metastases were found in the *EGFR* mutated group (*P* = 0.007, 0.004, and 0.043, respectively). Multivariable logistic regression analysis found that the model included age, gender, smoking history, air bronchogram, pleural tags, pleural and liver metastasis had a moderate predictive value for *EGFR* mutation status (AUC = 0.741, *P* < .0001). Exon-19 deletion was associated with air bronchogram which adjusted for age, gender and smoking history (*P* = 0.007, OR: 2.91, 95%CI: 1.25–7.79). The evidence of pleural tags, pleural and liver metastases go along with a higher probability of *EGFR* mutation in adenocarcinoma patients and air bronchogram is positively associated with Exon-19 deletion mutation.

## Introduction

Lung cancer remains the leading cause of cancer deaths for both men and women in the worldwide [1]. Many advances have been made in the understanding of the pathogenesis and management of lung cancer, particularly of adenocarcinoma (ADC). Specifically, the discovery of epidermal growth factor receptor (*EGFR*) mutations has changed lung cancer treatment. *EGFR* mutation are associated with a dramatic clinical response to the EGFR tyrosine kinase inhibitors (*EGFR* TKIs) gefitinib and erlotinib [2–4].

*EGFR* mutation testing is usually based on formaline fixed and paraffin embedded tumor specimens [5]. Approximately two thirds of non-small cell lung cancer (NSCLC) patients are diagnosed at an advanced stage of the disease [6] where only limited tumor specimens (biopsies, cytology) can be obtained in contrast to complete tumor resection. These limited tissue/cytologic samples are not always available or evaluable for diagnosis and mutation testing, leaving some patients unable to have the *EGFR* mutation status of their tumors determined [7]. Tumor heterogeneity [8] and the presence of lesions that are inaccessible to needle biopsy challenge the tumor biopsies as well. These challenges are accentuated in a later line setting because re-biopsy may not be feasible and tumor heterogeneity may be greater.

Therefore, a less invasive procedure to increase the pre-test probability for *EGFR* mutation analysis would be helpful. It has already been shown that non-smoking status, female and East Asian ethnicity are correlated with *EGFR* mutation, but they are not sufficient to select or exclude patients for *EGFR* mutation testing [9–10]. Several studies have demonstrated that circulating free tumor-derived DNA (ctDNA), which can be isolated from the plasma or serum of patients with NSCLC, is feasible to assess *EGFR* mutation status [11–12]. However, ctDNA analysis is technically challenging, the suitability and performance of ctDNA testing varies significantly between different geographic regions and different laboratories [13–14].

Computed tomography (CT) is wildly used in clinic to evaluate lung cancer patients and few studies have been carried out to investigate the imaging features of ADC with *EGFR* mutations. The results are controversial and the correlation of imaging features with *EGFR* mutation is unclear [15–18]. We hypothesize that there were some CT characteristics might correlate with *EGFR* mutation status and those CT characteristics might serve as a complementary way to suggest the *EGFR* mutation status.

In order to identify imaging characteristics of *EGFR* mutated ADC, we retrospectively analyzed computed tomography (CT) images of a cohort suffering from ADC with known *EGFR* mutation status.

## Materials and methods

This study was in compliance with the Health Insurance Portability and Accountability Act (HIPAA) regulations. Informed written consent for examinations (including CT, PET-CT and pathology examinations) were obtained from all patients. Clinical records of included patients suffering from ADC admitted to our 3rd level throacic hospital between February 2006 and October 2013 were reviewed retrospectively. The retrospective analysis was approved by the ethics committee of the medical school of the University of Heidelberg (IRB approval number S-048/2012). All patient records were anonymized and de-identified prior to analysis.

### Patients and clinical assessment

1575 consecutive ADC patients have been analyzed for *EGFR* mutation status (Exon 18–21), demographics and tumor histopathology. 271 patients (17%) showed *EGFR* mutations and a similar number of patients (n = 280) with *EGFR* wildtype from the same database was selected randomly for comparison. According to the inclusion criterion (available CT images before surgery, chemo- and radiotherapy in the Picture Archiving and Communication System, PACS [Synapse, Fuji Medical System]), 282 patients (male: female: 129: 153, mean age: 64 years) were included into the analysis. In a few cases (n = 6), the only pre-treatment imaging available was a positron emission tomogram with a non-enhanced CT (PET-CT) which was deemed adequate for lesion interpretation and characterization.

Gender, age, smoking status (non-smokers were defined as having smoked <100 cigarettes/life, former and active smokers were designated as smokers), malignant tumor history were retrieved from clinical documents (Table 1).

**Table 1:** Clinical characteristics of *EGFR* mutation (M) and wildtype (wt) cohorts.

Characteristics	*EGFR* mutation (n = 138)		*EGFR* wildtype (n = 144)		*P*
	No.	%	No.	%	
Age (years)					
Median	66		62		0.056
Range	33–87		40–84		
Sex					
Female	86	62%	67	47%	0.008*
Smoking status					
Non-Smokers	106	84%	94	67%	0.002*
N/A	11	8%	4	3%	
Malignant tumor history	19	15%	24	17%	0.628
N/A	11	8%	4	3%	
UICC stage^#^					0.066
I	4	3%	1	1%	
II	2	1%	5	4%	
III	24	18%	40	28%	
IV	104	78%	96	68%	
N/A	4	3%	2	1%	
N stage					0.983
N0	41	29%	41	30%	
N1	17	12%	17	12%	
N2	47	33%	42	30%	
N3	39	27%	38	28%	
Distant metastases					0.063
M0	30	22%	46	32%	
M1	104	78%	96	68%	
N/A	4	3%	2	1%	

Abbreviations: UICC: International Union Against Cancer; N/A: not applicable, LDH: lactat dehydrogenase

^#:^ 69 patients were staged by pathology and the rest of patients (n = 207) by clinical criteria.

*^:^ P<0.05 was considered as statistically significant.

### Histopathologic and *EGFR* analysis

Pathological diagnosis of the surgical specimens (n = 40), biopsies (n = 235) and cytological specimens (n = 7) were performed by board-certified pathologists according to the criteria of the 2004 WHO and 2011 IASLC/ATS/ERS classification [19]. *EGFR* mutations in exons 18–21 were determined by direct DNA sequencing as described previously in detail [20].

### CT evaluation

All included patients underwent chest CT or PET-CT, which had been conducted within one-month prior treatment and were interpreted on a PACS reading workstation retrospectively by two experienced chest radiologists (Z.J and D.J) in consensus blinded to the *EGFR* analysis results. CT examinations were performed at multiple institutions with a variety of helical scanners. The median section thickness used was 3mm (range from 0.5mm to 7mm).

**Fig 1:**
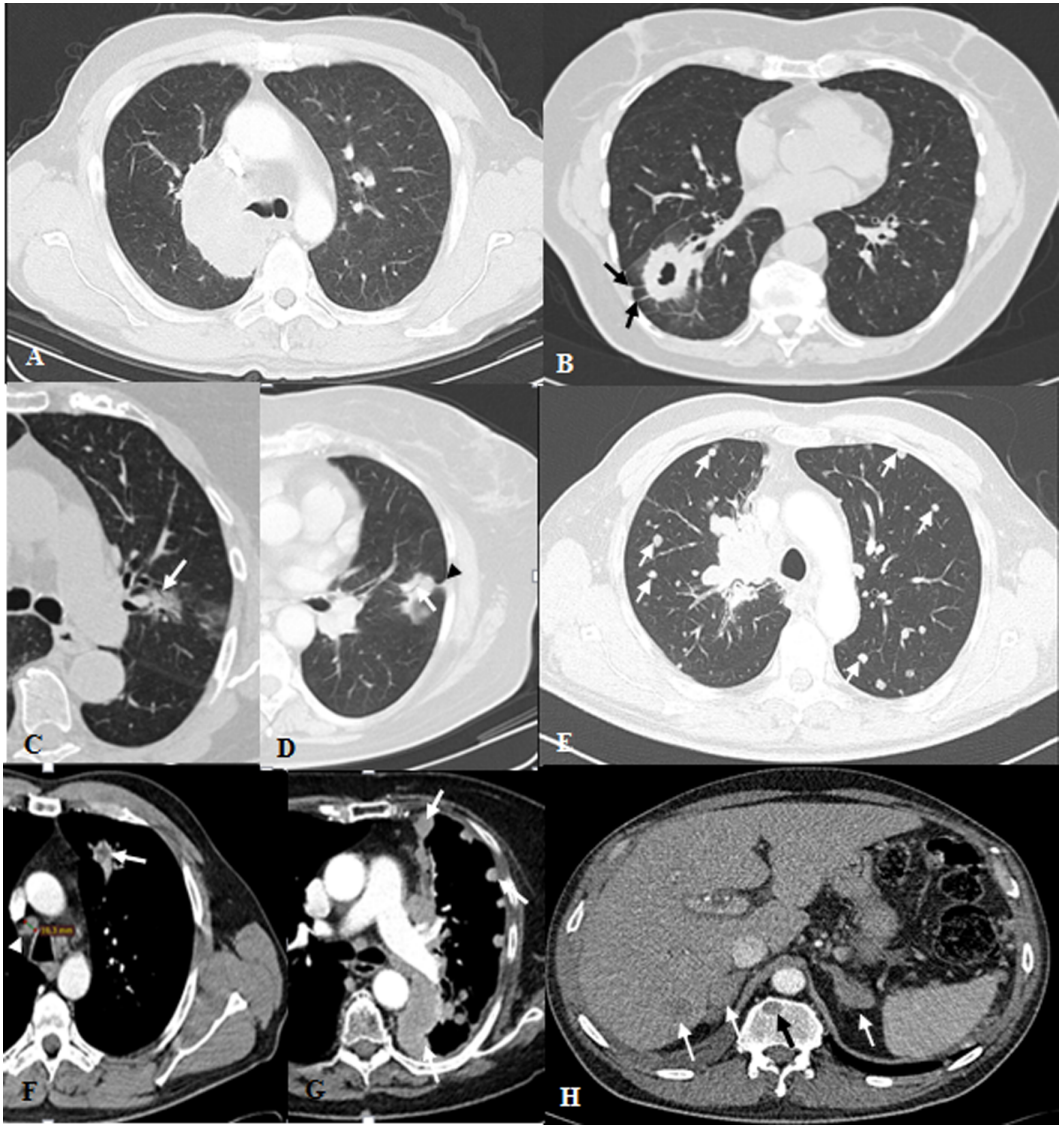
Transversal CT images of pulmonary adenocarcinoma with examples of the morphological features under investigation. (A) Central lesion with tumor-bearing lobe atelectasis; (B) peripheral cavitary solid mass with ovoid shape, predominant spiculated margins and associated pleural tags (arrow); (C) central irregular semi-solid lesion in smooth margin with air bronchogram (arrow), GGO account for 25%-50%; (D) peripheral lesion with irregular shape, air bronchogram (arrow) and pleural tag (arrowhead); (E) central solid lesion with irregular shape and predominant lobulated margins with bilateral pulmonary metastases (arrows); (F) Irregular shape tumor after contrast enhancement showed central necrosis (arrow) and contralateral mediastinal lymph node metastasis (arrowhead); (G) tumor-side pleural metastases (arrows); (H) liver, bone, and bilateral adrenal gland metastases (arrows).

Detailed imaging characteristics of the primary lesions, corresponding lymph nodes and distant metastases were recorded (Fig 1), including [21–26]:

1. Primary tumor: location (peripheral [outer perimeter within 1 cm of the pleura], middle and central); maximal axial size; shape (with or without atelectasis [obstructive, compressive and combining]; if without, the shape was classified as round, ovoid, lobulated or irregular); margin (smooth, spiculated, lobulated, concave and the predominant one); attenuation (solid, ground glass opacity [GGO], or semisolid with recording the percentage of GGO); presence of cavitation, air bronchogram (AB), calcification within the tumor; number of pleural tags; number of satellite nodules, including the maximum size of the biggest one; number of nodules in different ipsilateral lobes; pleural contacts (slight pleural contact, visceral pleural invasion, parietal pleural invasion); tumor enhancement (homogeneous, heterogeneous, large necrosis [more than 50% of the tumor area]).
2. Lymph node (LN): According to the 7th Edition TNM classification of lung cancer, LN were divided into three levels: N1 –N3). Short axial diameter of the biggest LN in each level was recorded if more than 5 mm as well as the attenuation of the corresponding LN after contrast enhancement (fatty, isodense, hyperdense, necrosis, mixed). A LN with a short axis diameter of more than 10mm was rated as metastasis [27–28].
3. Distant metastases: a) Presence of nodule(s) in the contralateral lung (recording the maximal axial diameter of the biggest nodule and the distribution of pulmonary metastases [regional or random and diffuse]); presence of lymphatic carcinomatosis or pleural carcinosis (all the pleural metastases were either proven by histology or clinical criteria (pleura with obvious irregular thickening or nodules of the pleura which became more irregular and thicker during the follow up); b) Nearly all included patients (n = 267/282) underwent additional imaging studies (abdominal CT or ultrasound, brain CT or magnetic resonance (MR) imaging and whole-body bone scanning with technetium 99m medronate) for tumor staging. All available images and reports were reviewed. The specific distant metastatic organ (brain, liver, adrenal gland etc), the number of corresponding metastases and the total number of distant metastases which were divided into two groups (less or more than five) were recorded.

### Statistical analysis

Initially, differences between categorical clinical and CT features were compared by χ2 test, Fisher’s exact test and Kruskal-Wallis test. Quantitative continuous variables in clinical and CT data were compared by Mann-Whitney-U test. Cases like “the size of satellite nodule and minor size of N1LN” with single missing data points too much were not included into the logistic regression analysis. Univariate logistic and multivariable logistic regression analysis which adjusted for age, gender and smoking history of two groups were employed to evaluate the relationship between clinical and CT features with *EGFR* mutation status. The optimized combination of different CT and clinical features to predicting the *EGFR* mutation status was performed by logistic regression analysis. *P* < 0.05 was considered to be statistically significant. Bonferroni adjustment method will be performed in multiple comparisons if necessary. The statistical software (SPSS 16.0; SPSS, Chicago, Ill) was used to perform the analysis and create graphs.

## Results

### *EGFR* mutation status and clinical characteristics

**Fig 2:**
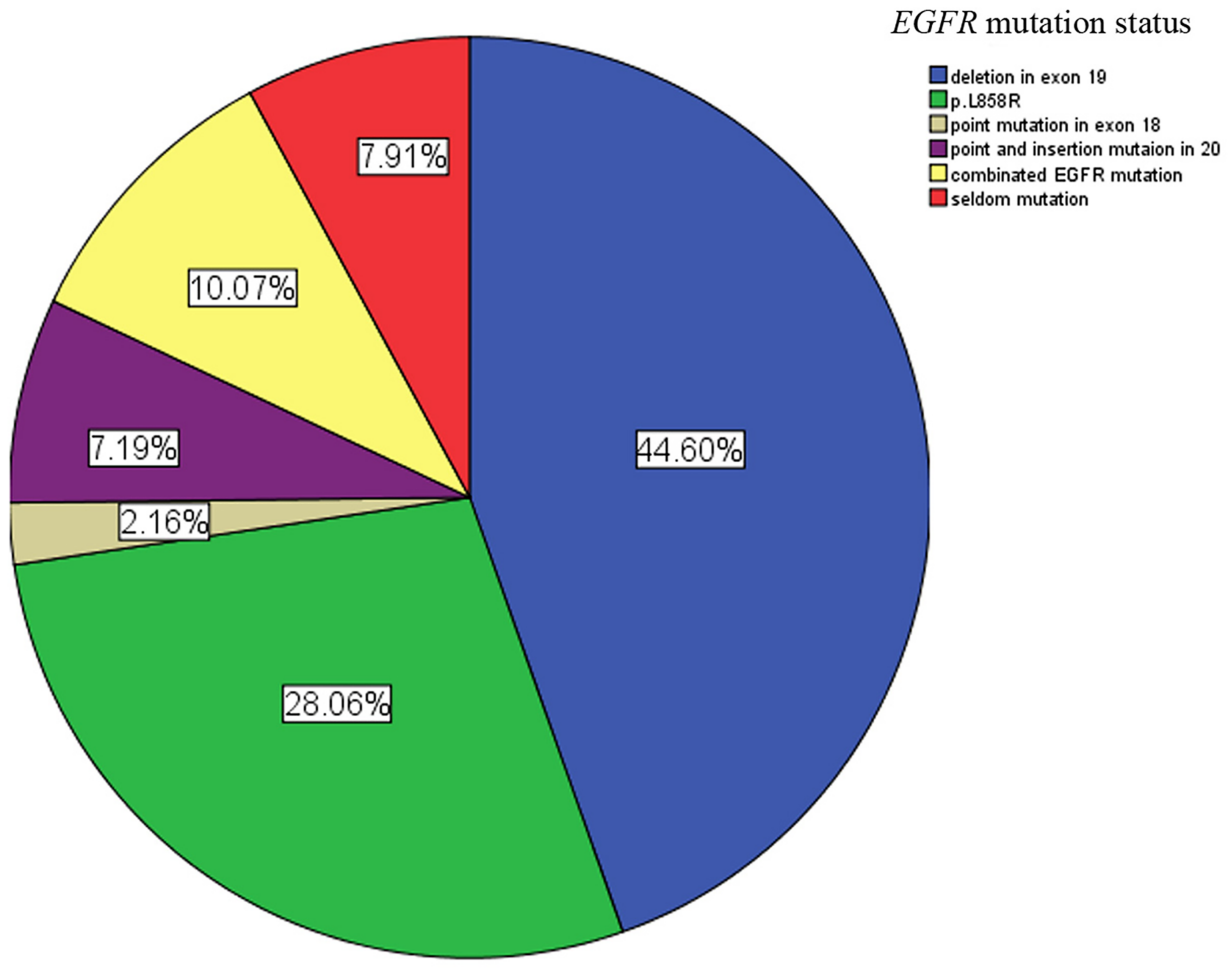
The percentage composition of the detected *EGFR* mutations. *Note*:*- Combined EGFR mutation stands for mutation at least was found in two exons (18–21)*, *for example point mutation in 18 and 20 exons*.*        - Seldom mutation means at least two combination of the way of EGFR mutation*, *such as deletion combined insertion in exon 19*.*        - There was one patients had both deletion in exon 19 and p*.*L858R*.

Our cohort included 282 patients. 138 patients (female: male: 86: 52, median age 66y) had *EGFR* mutations (M) while 144 patients (female: male: 67: 77, median age 62y) were *EGFR* wildtype (wt). The *EGFR* mutation group was constituted by 62 patients (45%) who had exon 19 deletions, 39 patients (28%) who harbored p.L 858R mutations and 27% of patients with other types of mutations (Fig 2).

All the clinical characteristics with *EGFR* mutation status were recorded in Table 1.

### *EGFR* mutation status and CT features

#### Primary tumor

No statistically significant differences between *EGFR* mutation status (mutation: M and wildtype: WT) in tumor location (peripheral, middle and central), size, shape, margin, attenuation, pleural contact and enhancement. However, AB was found in 44% (n_M_ = 55) of the *EGFR* mutated patients and 31% (n_WT_ = 39) of the patients with wt (*P* = 0.033). The percentage of *EGFR* mutated patients (n_M_ = 42 (33%) vs. n_WT_ = 26 (20%), *P* = 0.013) with more than one pleural tag was significantly higher. *EGFR* mutated patients showed a significantly higher incidence of satellite nodule (n_M_ = 71 [56%] vs. n_WT_ = 50 [38%], *P* = 0.004) while the median size of the biggest one was smaller (7mm vs. 9mm, *P* = 0.034), (Table 2) (S1 Table).

**Table 2:** Statistically significant imaging characteristics comparison between *EGFR* mutation statuses.

TNM	CT Features		*EGFR* wildtype (wt)			*EGFR* mutation (M)			P*	*P*^#^ (OR, 95%CI)
			Total	No.	%	Total	No.	%		
Primary tumor	Air bronchogram^1^		126	39	31%	125	55	44%	0.033	0.059
	Pleural tags (>1)^2^		133	26	20%	127	42	33%	0.013	0.007(2.27, 1.25–4.14)
	SN^3^	Number (≥1)	131	50	38%	126	71	56%	0.004	-
		Size(mm)	50	-	-	71	-	-	0.034	-
		Median	9	-	-	7	-	-	-	-
		Range	3–29	-	-	3–34	-	-	-	-
LN	Biggest N1^4^ LN size		90	-	-	88	-	-	0.023	-
	Median (mm)		14	-	-	12	-	-	-	-
	Range		7–33	-	-	6–22	-	-	-	-
Metastases	M1a	Pleural	144	30	21%	138	50	36%	0.004	0.004(2.30, 1.30–4.06)
	M1b	Liver^5^	140	8	6%	132	20	15%	0.010	0.043(2.54, 1.03–6.28)
		Bilateral adrenal^5^	140	9	6%	130	0	0%	0.002	NA

LN: lymph node, *EGFR*: epidermal growth factor receptor, SN: satellite nodules

OR: odd ratio, CI: confidence interval.

^1:^ 31 patients (M: wt = 13: 18) were excluded from this specific analysis, because the primary tumor of 10 patients could not be identified and the tumor-bearing lobe of the rest of patients were atelectasis, thus, the contour of the primary tumor was barely clearly recognized.

^2:^ 22 patients (M: wt = 11:11) were excluded from this specific analysis, because the primary tumor of 10 patients could not be identified and the tumor associated atelectasis hides the pleural relation of the tumor in these patients.

^3:^ Satellite nodules in 25 patients (M:wt = 12:13) could not be counted due to atelectasis and the not-identified primary tumors.

^4:^ N1: ipsilateral peribronchial and/or ipsilateral hilar LN and intrapulmonary nodes, only included the short size of LN more than 5mm.

^5:^ Information concerning liver and adrenal gland metastases was missing for 10 and 12 patients, respectively.

^#:^ The *P* value was calculated by multivariable logistic regression analysis which adjusted for age, gender and smoking history.

*^:^ *P* < 0.05 was considered as statistically significant.

#### Lymph nodes

The median short axis diameter of the biggest N1 LN in *EGFR* mutated patients was smaller (12 mm vs. 14 mm; *P* = 0.023) (Table 2). Other comparisons such as the short axis of the biggest LN in the other two levels and LN staging status were not statistically different.

#### Metastases

While random and diffuse, pulmonary metastases were more frequent in the *EGFR* mutation group (n_M_: 11 [8%] vs. n_WT_: 4 [3%], *P* = 0.052), this observation did not reach significance. The frequency of pleural metastases, of which 63 patients were confirmed by pathology and 17 patients were diagnosed according to clinical criteria, was higher in the *EGFR* mutation group (n_M_ = 50 [36%] vs. n_WT_ = 30 [21%], *P* = 0.004). With distant metastases, the overall incidence and the patients with more than five metastases were similar between the two groups. However, liver metastases were found significantly more often in *EGFR* mutated patients (*P* = 0.010) while bilateral adrenal gland metastases were found only in *EGFR* wt patients (*P* = 0.002). Interestingly, half of the adrenal metastases patients in *EGFR* wt group were manifested as bilateral metastases (Table 2) (S1 Table).

#### Univariate and multivariable logistic regression analysis

Univariate logistic analysis showed that gender, smoking history, AB, pleural tags, pleural metastasis and liver metastasis were significantly different in two groups and the *P* values were 0.008, 0.002, 0.033, 0.014, 0.004 and 0.013, respectively. (Table 2).

**Fig 3:**
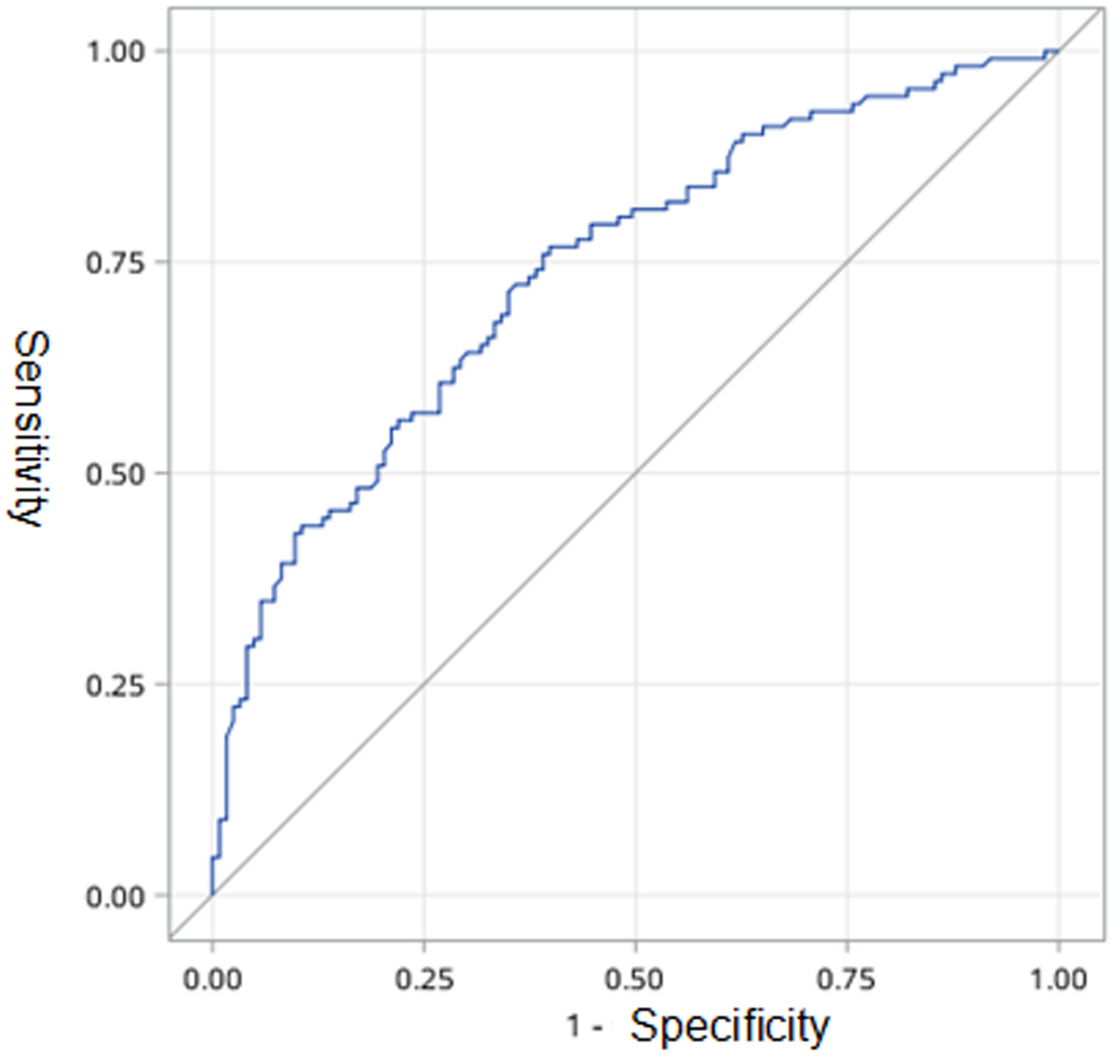
Receiver operator characteristic curve for the model which composed by age, gender, smoking history, air bronchogram, pleural tags, pleural and liver metastasis to predict *EGFR* mutation status. The area under the curve is 0.741.

Adjusted for age, gender and smoking history of two groups respectively, we found that the incidence of pleural tags (*P* = 0.007), pleural (*P* = 0.004) and liver metastases (*P* = 0.043) are significantly higher in *EGFR* mutated patients. (Table 2) However, the incidence of AB was nearly to show the significant difference in two groups (*P* = 0.059). Further, multivariable regression analysis found that combined age, gender, smoking history, AB, pleural tags, pleural and liver metastasis together which showed the highest predictive value (AUC = 0.741) (Fig 3).

### Association of the CT features with *EGFR* exon-19 deletion or p.L858R

After adjusting for age, gender and smoking history, multivariable logistic regression analysis showed that AB strongly associated with exon-19 deletion mutation (OR, 2.91; 95%CI: 1.25–7.79; *P* = 0.011), as compared to the rest of *EGFR* mutations. Whereas, compared with the rest of *EGFR* mutated patients, patients with p.L858R mutation had significant less AB (OR, 3.12; 95%CI: 1.25–7.79, *P* = 0.015) (Table 3). With other imaging features, there were no significant differences were found when associated them with *EGFR* exon-19 deletion or p.L858R.

**Table 3:** Within *EGFR* mutation group, significant correlations of deletion in exon 19 or p.L 858R with clinical and CT features.

Characteristics	Deletion in exon 19^1^				P*	*P* ^#^(OR, 95%CI)	p.L858R^1^				P*	*P* ^#^(OR, 95%CI)
	Yes	%	No	%			Yes	%	No	%		
Female (n = 138)	45/62	73%	41/76	54%	0.025	>0.05	23/39	59%	63/99	64%	0.611	-
Air bronchogram (n = 125)	33/59	56%	22/66	33%	0.011	0.007(2.91, 1.25–7.79)	9/34	27%	46/91	51%	0.016	0.015(3.12, 1.25–7.79)

OR: odd ratio, CI: confidence interval.

^#:^ The *P* value was calculated by multivariable logistic regression analysis which adjusted for age, gender and smoking history.

*^:^ *P* < 0.05 was considered as statistically significant.

^1:^ 13 patients were excluded out in this specific analysis, because the primary tumor of 5 patients could not be identified and the tumor-bearing lobe in 8 patients were atelectasis and the contour of the primary tumor was barely recognized.

## Discussion

This study has identified certain clinical and imaging characteristics which were correlated with *EGFR* mutations. According to previous reports [9, 10], we found that women and non-smokers tended to have *EGFR* mutation more often. To our knowledge, no study has previously been done with the objective of a comprehensive comparison of CT features of ADC patients with different *EGFR* mutation status, hence only single pattern such as GGO [16, 17, 29–32] have been evaluated so far. In addition, there were two studies have showed that ADC patients with malignant pleural effusion (MPE) had a higher incidence of *EGFR* mutation [33, 34].

Our study has showed that, several radiological features associated with *EGFR* mutation in ADC: the number of patients with AB, pleural tags, pleural and liver metastases was significantly higher if *EGFR* is mutated. Logistic regression analysis showed that the model composed by age, gender, smoking history, AB, pleural tag (n≥1), pleural and liver metastasis have a moderate predictive value for *EGFR* mutation. These could enable radiologists to better understand the imaging features which correlated with *EGFR* mutation and to applying this understanding into clinical practice by allowing radiologists to raise clinical suspicion for *EGFR* mutation.

Lepidic predominant ADC were already described to show AB frequently [35], while a correlation of *EGFR* mutations with the lepidic pattern has also been demonstrated [20]. This might explain why *EGFR* mutated patients in our cohort showed more AB. Koenigkam Santos M et al. [36] found that ADC was more commonly associated with pleural tags, compared to squamous cell carcinomas. In our study, mutated ADC had more pleural tags than wild type ADC.

Several reports [33, 37] found that overall survival (OS) could be prolonged in *EGFR* positive ADC patients with MPE undergoing *EGFR*-TKI therapy. Meanwhile, several retrospective studies [33, 34] discovered that patients with ADC and MPE had a higher rate of *EGFR* mutation. A functional variant of the *EGFR* promoter, 216G/T (rs712829), was associated with pleural spread of ADC which is usually accompanied with MPE [38]. This might explain the significantly higher number of pleural metastases in *EGFR* mutated patients. The accompanied high incidence of pleural effusion might cause more compressive atelectasis (n_M_ = 12 vs. n_WT_ = 6) in ADC with *EGFR* mutation.

In contrast to the previous findings [18, 39–41], neither significant differences in LN staging, pulmonary, nor brain metastases were noticed, while satellite metastases were significantly more frequent and of smaller size in *EGFR* mutated tumors. The latter findings were probably associated with the abundant angiogenesis of *EGFR* pathways [42] since this important mediator supports local spread resulting in multiple metastases in the same lobe or the whole lung. This mechanism might also explain the few necrosis in *EGFR* mutated tumors (n_M_ = 1; n_WT_ = 7, S1 Table). In addition, the short axis of N1 LN in *EGFR* mutated tumors was smaller. A possible explanation for this maybe *EGFR* was expressed in almost all cells with the only exception of the mature lymphohematopoietic cells [42].

The majority of detected *EGFR* mutations were either p.L858R or exon-19 deletions (39 + 62 = 101, 73%, Table 3). An association of CT features and OS with p.L858R or exon-19 deletions might be different. It has been demonstrated that p.L858R were correlated with the CT feature ‘invasive solid pattern’ [29], had not prolonged OS after *EGFR*-TKI therapy [43]. While exon-19 deletion, in contrast, had a longer progression-free survival (PFS) [44] and OS [43,45] after TKI treatment. Therefore, it is useful to know the correlation of significant clinical features and CT pattern with *EGFR* p.L858R or exon-19 deletions. As we found exon-19 deletion mutations were significantly more frequent in women and this was consistent with the finding that *EGFR* mutation favoring female patients. Even excitingly, we found exon-19 deletion is correlated with a significantly greater number of AB, whereas tumors with p.L858R mutation had significantly less frequent AB (Table 3).

Besides, our study has its own limitations. This was a retrospective study which would induce patient selection bias and the case included in the study were not enough, even we have already included as many cases as we could. However, these results might serve as a basement for our further international cooperative study and all those results still need be further validated by our prospective study.

## Conclusion

Clinical and CT-derived imaging characteristics are associated with activating *EGFR* mutations. Especially, the presence of AB, pleural tag, pleural and liver metastases may help to increase pretest probability for *EGFR* mutation. In addition, the presence of AB positively associated with *EGFR* exon-19 mutation.

## Supporting information

S1 Table Imaging characteristics comparison between different *EGFR* mutation status in primary tumor. (DOC)

S2 Table Imaging characteristics comparison between different *EGFR* mutation status in distant metastases (M). (DOCX)
