# Supplementary material for: CT characteristics in pulmonary adenocarcinoma with epidermal growth factor receptor mutation
Source: PLoS One. 2017 Sep 26;12(9):e0182741. doi: 10.1371/journal.pone.0182741 (PMC5614426; doi:10.1371/journal.pone.0182741)
Supplement: S1 Table — (DOC) [file pone.0182741.s001.doc]

Table A. Imaging Characteristics Comparison between Different *EGFR* Mutation Status in Primary tumor.

| CT Features | *EGFR* wildtype (wt) | | | *EGFR* Mutation(M) | | | *P* |
| --- | --- | --- | --- | --- | --- | --- | --- |
|  | Total | No | % | Total | No | % |  |
| Location | 144 |  |  | 138 |  |  | 0.745 |
| Peripheral |  | 59 | 41% |  | 54 | 39% |  |
| Middle |  | 41 | 29% |  | 46 | 33% |  |
| Central |  | 34 | 24% |  | 27 | 20% |  |
| Undeterminated |  | 10 | 7% |  | 11 | 8% |  |
| Size^1^ | 132 |  |  | 124 |  |  | 0.083 |
| Median(mm) |  | 41 |  |  | 39 |  |  |
| Range(mm) |  | 7 - | 140 |  | 12- | 120 |  |
| Shape | 144 |  |  | 138 |  |  | 0.062 |
| Round |  | 22 | 15% |  | 15 | 11% |  |
| Ovoid |  | 8 | 6% |  | 9 | 7% |  |
| Lobulated |  | 5 | 4% |  | 2 | 1% |  |
| Irregular |  | 90 | 63% |  | 76 | 55% |  |
| Undeterminated |  | 19 | 13% |  | 36 | 26% |  |
| Margin^2^ | 125 |  |  | 108 |  |  |  |
| Nonsmooth |  | 50 | 40% |  | 49 | 45% | 0.408 |
| Anysmooth |  | 75 | 60% |  | 59 | 55% |  |
| Nonspiculated |  | 75 | 60% |  | 60 | 56% | 0.493 |
| Anyspiculated |  | 50 | 40% |  | 48 | 44% |  |
| Nonlobulated |  | 68 | 54% |  | 58 | 54% | 0.915 |
| Anylobulated |  | 57 | 46% |  | 50 | 46% |  |
| Attenuation | 144 |  |  | 138 |  |  | 0.734 |
| Solid |  | 123 | 85% |  | 113 | 82% |  |
| Semi-solid |  | 9 | 6% |  | 9 | 7% |  |
| GGO |  | 0 | 0% |  | 1 | 1% |  |
| Undeterminated |  | 12 | 8% |  | 15 | 11% |  |
| Cavitation | 144 |  |  | 138 |  |  | 0.500 |
| No |  | 112 | 78% |  | 115 | 83% |  |
| Yes |  | 14 | 10% |  | 10 | 7% |  |
| Undeterminated |  | 18 | 12% |  | 13 | 10% |  |
| Calcification | 144 |  |  | 138 |  |  | 0.812 |
| No |  | 122 | 85% |  | 118 | 86% |  |
| Yes |  | 12 | 8% |  | 9 | 7% |  |
| Undeterminated |  | 10 | 7% |  | 11 | 8% |  |
| Noudules in different  ipsilateral lobe | 144 |  |  | 138 |  |  | 0.631 |
| No |  | 77 | 54% |  | 66 | 48% |  |
| Yes |  | 59 | 41% |  | 64 | 46% |  |
| Undeterminated |  | 8 | 6% |  | 8 | 6% |  |
| Pleural contact | 144 |  |  | 138 |  |  | 0.758 |
| No pleural contact |  | 28 | 19% |  | 19 | 14% |  |
| Slightly pleural contact |  | 21 | 15% |  | 22 | 16% |  |
| Invasion of VP |  | 57 | 40% |  | 59 | 43% |  |
| Invation of PP |  | 25 | 17% |  | 23 | 17% |  |
| Undeterminated |  | 13 | 9% |  | 15 | 11% |  |
| Tumor enhancement^3^ | 144 |  |  | 137 |  |  | 0.068 |
| Homogeneous |  | 21 | 15% |  | 28 | 20% |  |
| Heterogeneous |  | 88 | 61% |  | 74 | 54% |  |
| Large necrosis |  | 7 | 5% |  | 1 | 1% |  |
| Contrast(-) and  Undeterminated |  | 28 | 19% |  | 34 | 25% |  |

VP: Visceral Pleural; PP: Parietal Pleural

1: 28 patients (M: wt=14: 12) were excluded from this specific analysis, because the primary tumor of 10 patients could not be identified clearly and the tumor-bearing lobe of the rest of patients were atelectasis.

2: 49 patients (M: wt=30: 19) were excluded from this specific analysis, because the primary tumor of 10 patients could not be identified and the tumor-bearing lobe of the rest of patients were atelectasis, thus, the contour of the primary tumor was barely recognized.

3. One GGO nodule in EGFR mutation group was excluded in this specific analysis.
