# Supplementary material for: CT characteristics in pulmonary adenocarcinoma with epidermal growth factor receptor mutation
Source: PLoS One. 2017 Sep 26;12(9):e0182741. doi: 10.1371/journal.pone.0182741 (PMC5614426; doi:10.1371/journal.pone.0182741)
Supplement: S2 Table — (DOCX) [file pone.0182741.s002.docx]

Table B. Imaging Characteristics Comparison between Different *EGFR* Mutation Status in Distant Metastases (M).

| CT Features | *EGFR* wildtype (wt) | | | EGFR Mutation (M) | | | *P* |
| --- | --- | --- | --- | --- | --- | --- | --- |
|  | Total | No. | % | Total | No. | % |  |
| Contralateral lobe  nodules | 144 |  |  | 138 |  |  | 0.774 |
| No |  | 75 | 52% |  | 66 | 48% |  |
| Yes |  | 68 | 47% |  | 71 | 51% |  |
| Undeterminated |  | 1 | 1% |  | 1 | 1% |  |
| Pulomonary M-Random and diffuse | 144 |  |  | 138 |  |  | 0.052 |
| No |  | 4 | 3% |  | 11 | 8% |  |
| Yes |  | 140 | 97% |  | 127 | 92% |  |
| Lymphangiosis M | 144 |  |  | 138 |  |  | 0.248 |
| No |  | 133 | 92% |  | 119 | 86% |  |
| Yes |  | 7 | 5% |  | 12 | 9% |  |
| Undeterminated |  | 4 | 3% |  | 7 | 5% |  |
| Number of distant M | 140 |  |  | 127 |  |  | 0.123 |
| ≤5 |  | 124 | 89% |  | 104 | 82% |  |
| >5 |  | 16 | 11% |  | 23 | 18% |  |
| N/A | 4 |  |  | 11 |  |  |  |
| Adrenal M | 140 |  |  | 130 |  |  | 0.462 |
| No |  | 122 | 87% |  | 117 | 90% |  |
| Yes |  | 18 | 13% |  | 13 | 10% |  |
| N/A | 4 |  |  | 8 |  |  |  |
| Brain M | 140 |  |  | 127 |  |  | 0.646 |
| No |  | 115 | 82% |  | 107 | 84% |  |
| Yes |  | 25 | 18% |  | 20 | 16% |  |
| N/A | 4 |  |  | 11 |  |  |  |
| Bone M | 140 |  |  | 129 |  |  | 0.101 |
| No |  | 106 | 76% |  | 86 | 67% |  |
| Yes |  | 34 | 24% |  | 43 | 33% |  |
| N/A | 4 |  |  | 9 |  |  |  |

N/A: not applicable, due to non-adequate imaging and clinical information.
